# Successful Medical Weight Loss in a Community Setting

**DOI:** 10.4172/2165-7904.1000248

**Published:** 2015-02-27

**Authors:** Douglas Carney, Stephen Schultz, Jeong Lim, William Walters

**Affiliations:** 1 Weight Management Program, Salem, Oregon, USA; 2 Health & Nutrition Technology Inc., Carmel, California, USA; 3 Oregon Health & Science University, Portland, Oregon, USA

**Keywords:** Low-calorie diet, Meal replacements, Obesity treatment, Weight loss, Weight maintenance

## Abstract

**Background:**

Research has shown that meal replacement calorie reduction combined with lifestyle change can more than double the weight loss seen with other diets. However, its widespread acceptance by physicians has been limited, perhaps waiting on evidence that patients are successful in keeping lost weight off.

**Methods:**

Obese patients (108.4 ± 25.7 kg, BMI 38.1 ± 7.9 kg/m^2^) used a diet of meal replacements combined with weekly classes. While learning about nutrition, exercise, and accountability, patients tracked calorie intake and physical activity. Weight loss and retention rates for rapid weight loss and maintenance phases were measured. Weights then obtained years after treatment ended showed that patients were keeping lost weight off without any ongoing clinic intervention.

**Results:**

Records of 714 patients treated in a medical weight loss practice from 2004 through 2012 were reviewed. For all patients, weight loss was 13.6 ± 8.3 kg, and 12.5% of initial weight. The 469 patients who completed 16 weeks of weight loss classes lost 16.7 ± 7.2 kg and 15.1%. 433 patients then enrolled in maintenance classes, and after 12 months had regained only 0.1 ± 9.1 kg and 0.4%. Follow up weights obtained from 173 patients more than 2 years after treatment ended showed persisting weight loss of 14.3 ± 13.7 kg and 12.9%. Final BMI was 32.7 ± 7.7 kg/m^2^.

**Conclusion:**

In a medical weight loss program that used meal replacements to reduce calorie intake combined with weekly behavior change classes, weight loss was 16.2 kg and 14.4% for the 61% of all enrollees who completed 16 months of treatment. More importantly, over 2 years later, weight loss of 14.3 kg and 12.9% of initial weight persisted, and patients were not regaining their lost weight.

## Introduction

As the obesity epidemic persists, middle-aged persons are gaining 10 pounds per decade. For U.S. adults, 69.2% now have Body Mass Indexes (BMI’s) over 25 and 35.9% are obese [1–3]. A wealth of data demonstrates adverse health effects of being overweight. Further, costs for obesity and associated chronic disease amount to 10% of health care spending, which for obese people is $1429/year more than for those with normal weights [4]. Such high burdens and costs mean that patients continue to need effective treatment.

In response, funding for obesity research has increased to nearly $1 billion annually, but only limited and temporary weight loss is reported in many trials [5]. Much research has focused on testing diets differing in macronutrients that show little differences in weight loss. Individual acceptance of overweight as the norm, and resistance of physicians to intervene despite some modest research success with weight loss also slows our efforts to deal with obesity [6–8].

Bariatric surgery results are encouraging, with 25% loss at up to 6 years. But complications and high costs suggest this will not be the treatment chosen for most obese persons [9]. The mainstay of treatment for most remains lifestyle change aimed at reducing caloric intake and increasing physical activity [10,11].

Research shows that medical interventions using meal replacements can also lead to significant weight loss. The Look AHEAD Research Group used reduced calorie intake including meal replacements, and lifestyle change (counseling and increased physical activity). They reported weight loss of 6% at 9.6 years [12]. Rock and colleagues used commercially prepared meals combined with counseling to achieve 7.9% loss at 2 years [13]. Goodpaster et al. used calorie reduction including meal replacements with lifestyle counseling and showed 10.9% loss at 1 year [14]. The STEP diet of meal plans (including meal replacements) and counseling reported 8.1% loss at 18 months [15]. The LOSS study of extremely obese patients used meal replacements and intensive medical intervention in achieving 9.7% loss at 2 years [16].

This research demonstrates that calorie reduction using meal replacements combined with lifestyle change is effective for weight loss during several years of treatment. Community-based efforts that apply these findings, improve on their results, and learn whether lost weight is kept off after treatment are needed [17].

Such studies can rely on proven facts about obesity treatment that do not require more research. Such facts include that use of meal replacements promotes greater weight loss; that rapid weight loss is more effective than slow weight loss; and that continuing the calorie reduction and lifestyle that achieved the weight loss promotes the maintenance of lower weight long term [18]. We apply these known treatment facts in a community medical practice setting to increase total weight lost, and show that patients can keep lost weight off.

## Methods

Most medical interventions are effective only while treatment continues. Similarly most medical weight loss hasn’t been successful long term, as weight gain often occurs when treatment ends. We report a medical intervention with high program completion rates that not only leads to substantial weight loss during treatment, but also persistent weight loss years after treatment ends.

All 714 patients enrolled in a medically supervised obesity treatment clinic between 2004 and 2012 were included in this clinical series. They used Health One meal replacement and the Program for Health and Weight Management (Health and Nutrition Technology, Inc., Carmel, CA).

After patients were evaluated by a physician and gave informed consent, they chose a realistic goal weight (BMI<30), and underwent 16 weeks of rapid weight loss. They used a hypo caloric meal replacement diet (800 kcal; 75 g protein, 110 g carbohydrate, 5 g fat), plus food additives to provide 900 kcal/day, while attending weekly classes. Participants were taught to record daily calorie intake, calories burned during physical activity, fluid intake (goal 2 liters/day), and behavioral successes, then report results during class. Health educator focus was on nutrition basics and calorie counting, and how to self monitor and be accountable. Patients gradually increased physical exercise to ≥60 minutes/day and were taught to convert exercise minutes to calories with a goal ≥2000 kcal/week of physical activity (PA) calories burned. After initial weight loss, patients entered a year of maintenance classes to practice these skills. Meal replacement (MR) diet continued to goal weight followed by transition to a low processed carbohydrate diet using a high intake of fruits, vegetables, and whole foods with optional MR as needed to maintain daily calorie intake ≤10 kcal/lb. Patients paid monthly for class sessions. MR purchases were extra and considered a replacement cost since they took the place of grocery store food.

Attempts were made to contact all patients up to several years after last clinic visit. For those who responded, follow up weights were obtained to assess the natural history of weight change following this treatment.

Data on individual weight loss and program completion rates were compiled from medical records and analyzed by the Biostatistics and Design Program (BDP) at Oregon Health & Science University (OHSU). BDP also obtained ethical review and approval of this study from OHSU’s Institutional Review Board (IRB).

## Results

Values are reported as mean±standard deviation. The 714 enrollees weighed 108.4±25.7 kg with BMI’s 38.1±7.9 kg/m^2^. Age was 50.5±14.6 years; 75% were women. Men were slightly older and slightly heavier than women. Other demographic characteristics (such as race, ethnicity, or income level) were not obtained. Baseline characteristics, weight change, and retention rates are shown in Table 1. It includes Weight Loss phase, Maintenance phase, Follow-up after patients left treatment, and Overall weight change.

For all Weight Loss enrollees (N=714), loss was 13.6±8.3 kg, and 12.5±6.1% of initial weight. Completers were 469 patients (2/3 of all enrollees) who continued through 16 weeks of rapid weight loss classes. They lost 16.7±7.2 kg, 15.1±5.3% of initial weight, and BMI decreased to 32.7 ± 6.7 kg/m^2^. Maintenance patients were 433 enrollees who attended maintenance classes. They had a weight gain of only 0.1±9.1 kg, and 0.4±9.4% after 12±14.5 months. At the end of treatment (Weight Loss plus Maintenance) loss of 16.2 kg and 14.4% persisted, and BMI was 33.0±7.2 kg/m^2^.

To learn if our patients were having long-term success, we obtained follow-up weights for 173 patients (40% of those who completed treatment) 29.1 ± 22.2 months after treatment ended. Final Overall weight loss was 14.3±13.7 kg and 12.9±11.3% of initial weight. BMI was 32.7±7.7 kg/m^2^.

**Figure 1:**
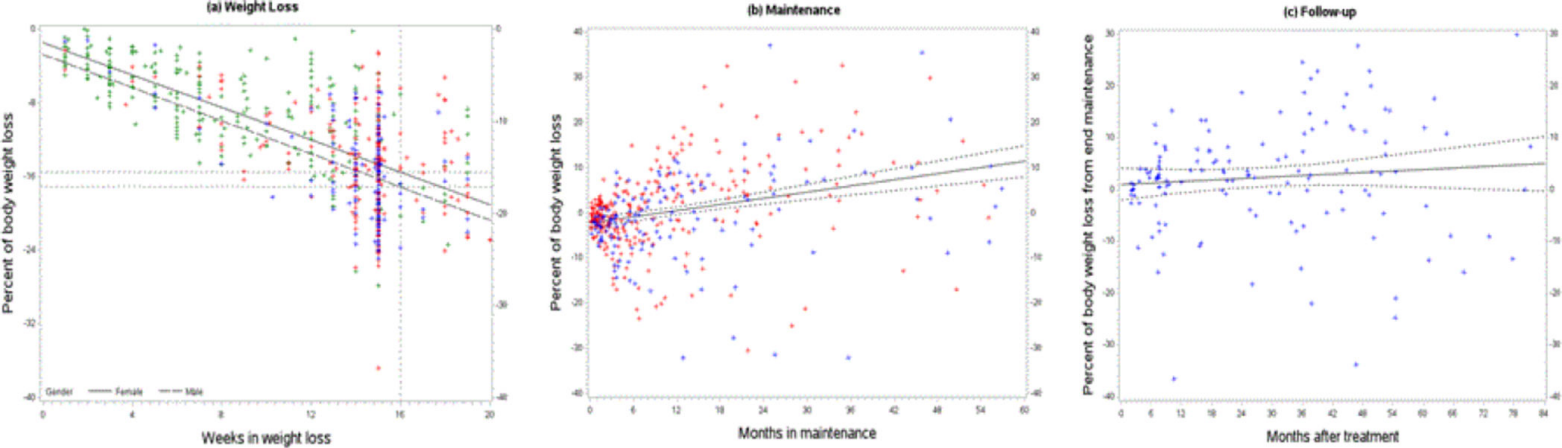
Weight Change in Weight Loss, Maintenance, and Follow-up phases.

In Figure l. individual data points are plotted with regression lines showing percent of body weight lost by time spent in each phase. During Weight Loss (Figure 1a), weight change is linear, with loss of 15% of initial weight for women and 16% for men at 16 weeks. (β=-0.88, p<0.0001). For Maintenance (Figure 1b), patients are asked to stay in classes for at least 12 months. Weight gain during that time is 2.6% per year (β=0.22, p<0.0001). The plot of Follow-up data (Figure 1c) shows the natural history of weight change for patients after they leave treatment. The regression line suggests that little further weight gain is occurring (β=0.05, p=0.26).

Individual data points are plotted with regression lines showing percent of body weight lost by time spent in each phase. Individuals who participated only in Weight Loss are shown in green; those in Weight Loss and Maintenance are in red; those with Follow-up are in blue.

**Figure 2:**
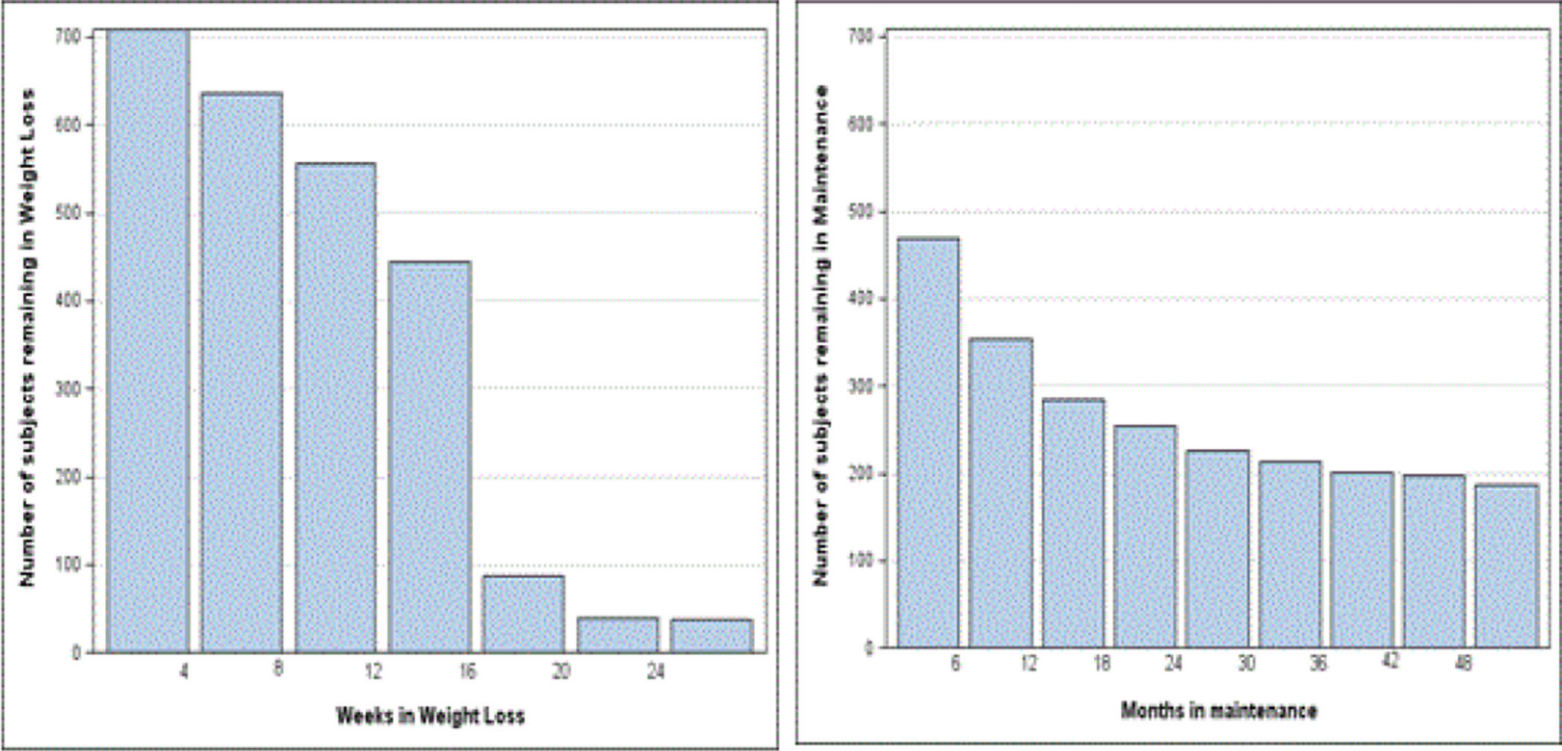
Attrition rates. Subjects remaining in weight loss (left) and maintenance (right) over time.

Figure 2 shows how the number of patients in Weight Loss and Maintenance declines over time. Of all 714 enrollees, 469 (2/3 of enrollees) complete 16 weeks of rapid Weight Loss, and 433 (61% of enrollees) participate in Maintenance.

## Discussion

A general medical view is that patients have few options for significant weight loss, and that most weight lost by calorie restriction and lifestyle change is regained within 5 years.

It is noteworthy that our patients are keeping their lost weight off.

In an earlier clinical series of 917 patients enrolled between 1991 and 2004, we show 14% average loss after 2 years of treatment using meal replacement technology and intensive lifestyle classes [19]. However only 10% of patients were still being seen, and no follow up weights were later obtained to show the natural history of weight change after leaving our program.

In this series, 61% of enrolled patients completed 16 months of treatment (Weight Loss plus Maintenance) with 16.2 kg and 14.4% loss, and 40% of completers were maintaining a 14.3 kg and 12.9% loss almost 2 ½ years after treatment ended. This shows our success in translating research results into a program with greater average weight loss, more patients completing treatment, and evidence of long-term success in keeping lost weight off. Stable reduced weight and body mass in our patients likely reflect their adoption of new habits based on known facts about obesity treatment. They learned that using meal replacements to reduce calorie intake is effective; and that maintaining weight loss requires continued commitment to behaviors that caused the loss including calorie reduction, daily exercise and record keeping. Our instructors also teach a whole food approach to food selection during Maintenance similar to that used by Bazzano et al. in their successful low processed carbohydrate diet trial [20]. All of these factors may contribute to our patients sustained weight loss.

Research on use of meal replacements and lifestyle change has shown it to be a medical model for reasonable weight loss. We were able to successfully translate this research into a program that is medically supervised and located in a community setting. We were able to improve average weight loss by about 50% over that seen in recent meal replacement research, and we had excellent patient retention rates rarely seen outside a controlled research setting. Ours is also one of the first non-surgical studies to look at weight loss more than 2 years after treatment, and we show that those completing this intensive medical program are keeping their lost weight off.

Limitations include that only 40% of treatment completers could be reached for follow-up weights, and long-term success for the others isn’t known. Unknown also is whether results from these highly motivated patients can be generalized to more of the obese population. As incentives and insurance coverage for dealing with obesity become more common, such questions may be clarified.

## Conclusion

In a study of 714 obese patients (108.4 ± 25.5 kg, BMI 38.17.9 kg/m^2^) enrolled in a medical weight loss program that used meal replacements to reduce calorie intake combined with behavior change classes, 61% of patients maintained a 16.2 kg and 14.4% weight loss after 16 months of Weight Loss and Maintenance treatment. More importantly, in an era where obesity is often considered intractable, 40% of patients who completed this program were successfully maintaining their weight loss. Over 2 years after last treatment ended, loss of 14.3 kg and 12.9 % of initial weight persisted, and patients were not regaining.

## Figures and Tables

**Table 1:** Baseline characteristics (weight change during the weight loss, maintenance, and follow-up phases, overall weight change).

		All	Women	Men	Completed≥13 weeks	Participated in maintenance	Follow-up
N		714	534	180	469	433	173
Age (years)		50.5 ± 14.6	49.6 ± 14.5	53.1 ± 14.5	51.0 ± 14.3	51.3 ± 14.1	52.9 ± 17.8
Height (cm)		168.5 ± 9.9	164.6 ± 7.5	179.9 ± 7.1	168.9 ± 10.1	168.4 ± 9.5	168.5 ± 9.5
**Weight Loss**							
Duration (weeks)		13.1 ± 7.9	12.7 ± 6.8	14.4 ± 10.4	16.5 ± 7.5	15.1 ± 6.7	14.6 ± 7.2
Onset:	Weight (kg)	108.4 ± 25.7	101.9 ± 22	127.7 ± 26.2	110.0 ± 24.9	108.9 ± 25.3	107.9 ± 26.8
	BMI (kg/m^2^)	38.1 ± 7.9	37.7 ± 7.8	39.5 ± 8.0	38.5 ± 7.6	38.4 ± 7.9	38 ± 8.4
End	Weight (kg)	94.8 ± 22.7	89.7 ± 19.9	110 ± 52.9	93.2 ± 21.5	92.6 ± 21.9	91.9 ± 22.7
	BMI (kg/m^2^)	33.4 ± 7.1	33.2 ± 7	34.1 ± 7.3	32.7 ± 6.7	32.6 ± 6.9	32.3 ± 7.5
Loss (kg)		−13.6 ± 8.3	−12.2 ± 7.6	−17.7 ± 9.1	−16.7 ± 7.2	−16.3 ± 7.2	−16.1 ± 7.6
Percent of loss (%)		−12.5 ± 6.1	−12.0 ± 6	−13.8 ± 6.3	−15.1 ± 5.3	−14.8 ± 5.4	−.7 ± 5.6
**Maintenance**							
Duration (months)						12 ± 14.5	13.7 ± 15.2
Onset	Weight (kg)					93.6 ± 22.6	93 ± 24.6
	BMI (kg/m^2^)					32.9 ± 7	32.5 ± 7.9
End	Weight (kg)					93.7 ± 23.2	92.1 ± 25.3
	BMI (kg/m^2^)					33.0 ± 7.2	32.2 ± 7.8
Loss (kg)						0.1 ± 9.1	−0.8 ± 8.6
Percent of loss (%)						0.4 ± 9.4	−0.7 ± 9.9
**Follow-up**							
Duration (months)							29.1 ± 22.2
End	Weight (kg)						93.6 ± 25.2
	BMI (kg/m^2^)						32.7 ± 7.7
Loss (kg)							1.7 ± 10.5
Percent of loss (%)							2.4 ± 11.0
Overall							
Duration (months)							46.1 ± 24.8
Loss (kg)							−14.3 ± 13.7
Percent of loss (%)							−12.9 ± 11.3

## Abbreviations

BMI: Body Mass Index
PA: Physical Activity
MR: Meal Replacement

## References

[1] Morrato EH, Allison DB (2012). FDA Approval of Obesity Drugs. JAMA.

[2] Hennekens CH, Andreotti F (2013). Leading Avoidable Cause of Premature Deaths Worldwide: Case for Obesity. Am J Med.

[3] Flegal KM, Carroll MD, Kit BK, Ogden CL (2012). Prevalence of Obesity and Trends in the Distribution of Body Mass Index Among US Adults, 1999–2010. JAMA.

[4] Kushner RF (2010). Tackling Obesity. Is Primary Care Up to the Challenge?. Arch Intern Med.

[5] Hebert JR, Allison DB, Archer E, Lavie CJ, Blair SN (2013). Scientific Decision Making, Policy Decisions, and the Obesity Pandemic. Mayo Clin Proc.

[6] Alpert JS (2010). Combating the Obesity Epidemic in the United States. Am J Med.

[7] Pagoto SL, Appelhans BM (2013). A Call for an End to the Diet Debates. JAMA.

[8] Johnston BC, Kanteus S, Bandayrel K, Wu P, Naji F (2014). Comparison of Weight Loss Among Named Diet Programs in Overweight and Obese Adults. JAMA.

[9] Wolfe BM, Purnell JQ, Belle SH (2013). Treating Diabetes With Surgery. JAMA.

[10] Livingston EH, Zylke JW (2012). Progress in Obesity Research. JAMA.

[11] Wing RR (2010). Treatment Options for Obesity. JAMA.

[12] Look AHEAD Research Group (2013). Cardiovascular Effects of Intensive Lifestyle Intervention in Type 2 Diabetes. N Engl J Med.

[13] Rock CL, Flatt SW, Sherwood NE, Karanja N, Pakiz B (2010). Effect of a Free Prepared Meal and Incentivized Weight Loss Program on Weight Loss and Weight Loss Maintenance in Obese and Overweight Women. JAMA.

[14] Goodpaster BH, DeLany JP, Otto AD, Kuller L, Vockley J (2010). Effects of Diet and Physical Activity Interventions on Weight Loss and Cardiometabolic Risk Factors in Severely Obese Adults. JAMA.

[15] Jakicic JM, Tate DF, Lang W, Davis KK, Polzien K (2012). Effect of a Stepped-Care Intervention Approach on Weight Loss in Adults. JAMA.

[16] Ryan D, Johnson WD, Myers VH, Prather TL, McGlone MM (2010). Nonsurgical Weight Loss for Extreme Obesity in Primary Care Settings. Arch Intern Med.

[17] Rodgers GP, Collins FS (2012). The Next Generation of Obesity Research. JAMA.

[18] Casazza K, Fontaine KR, Astrup A, Birch LL, Brown AW (2013). Myths, Presumptions, and Facts about Obesity. N Eng. J Med.

[19] Carney DM, Schultz SR, Carney SM (2009). Medical Obesity Treatment: Long-Term Success in a Primary Care Setting. J Diabetes Sci Technol.

[20] Bazzano LA, Hu T, Reynolds K, Yao L, Bunol C (2014). Effects of Low-Carbohydrate and Low-Fat Diets. Ann Intern Med.

